# Analysis of parental abnormal chromosomal karyotype and subsequent live births in Chinese couples with recurrent pregnancy loss

**DOI:** 10.1038/s41598-021-98606-4

**Published:** 2021-10-13

**Authors:** Shan Li, Mei Chen, Peng-Sheng Zheng

**Affiliations:** 1 Department of Reproductive Medicine, The First Affiliated Hospital of the Medical College, Xi’an Jiaotong University, 76 West Yanta Road, Xi’an, 710061 Shaanxi Province People’s Republic of China; 2 Section of Cancer Stem Cell Research, Key Laboratory of Environment and Genes Related To Diseases, Ministry of Education of the People’s Republic of China, Xi’an, 710061 Shaanxi People’s Republic of China

**Keywords:** Cytogenetics, Chromosome abnormality, Genetic translocation

## Abstract

The frequency and distribution of chromosomal abnormalities and the impact of parental chromosomal aberration on the pregnancy outcomes of couples with recurrent pregnancy loss remains controversial. 3235 RPL couples who experienced two or more miscarriages before 20 weeks were diagnosed in our tertiary referral hospital during 2008–2018 and included in the single-center retrospective cohort study covering a 10-year period. Chromosome aberration was detected in 121 (3.74%) among 3235 RPL couples which included 75 female and 46 male cases at an individual level. 101 cases were structural aberrations including balanced translocations in 46(38.0%) cases, Robertsonian translocations in 13(10.7%) cases, inversions in 42(34.7%) cases and 20(16.5%) cases were numerical aberrations. 121 carriers and 428 non-carriers were followed up for two years, 55 carriers and 229 non-carriers were subsequent pregnant after diagnosis by natural conception or intrauterine insemination. The frequency of carriers to have a health newborn was not significantly different with non-carriers (72.7% vs. 71.2%, adjusted *P* = 0.968). This study described the majority of carriers were balanced translocations and chromosome aberrations had a limited influence on live birth rate from the present data. The results of the study also remind us that natural conception may be also a good alternative rather than PGD (Pre-implantation Genetic Diagnosis) which is common in many other reproductive centers for such patients.

## Introduction

Recurrent pregnancy loss (RPL) is defined by the ESHRE guidelines in November 2017 as the loss of two or more pregnancies^1^. According to the history of live birth, it can be divided into primary and secondary RPL^2^. The causes of RPL are very complicated. In addition to anatomy, endocrine, thrombophilic, immune and other factors, embryo chromosomal abnormalities are often considered an important cause of miscarriage^3,4^. The embryo chromosomal abnormality rate in the general population is 60%^5^, while the rate in the recurrent miscarriage is 29–60%^6–8^. Embryonic chromosomal abnormalities may occur during the mitosis of embryo development, or come from parental abnormal ovum or sperm. For example, parental balanced chromosomes lead to unbalanced gametes which might cause abortion^9^. Therefore, the chromosomal karyotype of both parents is considered to be an important examination in the cause of recurrent miscarriage recommended by American Colleges of Obstetricians and Gynecologists^10^. However, the evidence that parental chromosomal abnormalities lead to miscarriage is still unclear, a considerable percentage of couples with chromosomal abnormalities have successfully given birth^11^. In addition to chromosomal factors, other factors may cause miscarriages that coexist with chromosomal aberrations. Due to the limited number of samples in couples with abnormal chromosomes, other confounding pathological factors such as immune and endocrine problems could not be excluded in this study. Therefore, it is more difficult to judge and analyze the cause of miscarriage due to parental chromosomal abnormalities, which often makes clinicians' understanding of parental chromosomal abnormalities leading to miscarriage not accurate enough.

The study attempts to summarize the frequency of abnormal chromosomal karyotype couples, the topography of abnormal types, and the frequency of the male and female carriers in the recurrent miscarriage population. The coexistence of other causes of miscarriage and respective pregnancy outcomes were further evaluated.

## Materials and methods

### Study population

The study was approved of the Ethic Committee of The First Affiliated Hospital of Xi’an Jiaotong University according to the declaration of Helsinki. All the participates were informed and signed consent for the study. From January 2008 to December 2018, a total of 5680 couples who had two or more spontaneous miscarriage before the 20th week of pregnancy came to our reproductive center for outpatient treatment. The included patients must provide objective evidence of past birth history, including HCG testing, or the histology report after curettage and evacuation of uterine or the gestational sac under ultrasound, all the clinical data bring to the study was carefully recorded and checked.

### Etiological screening investigation

All the patients were also recommended to investigate some presumptive causes of abortion beside chromosome analysis, such as Mycoplasma and Chlamydia infection, B mode ultrasound for uterine anatomical structure (including arcuate uterus, septate uterus, bicornuate uterus, naive uterus and intrauterine adhesions, endometrial polyps, uterine fibroids, adenomyosis), flow cytometry for peripheral blood lymphocyte subsets including natural killer cellsets and regulatory T cell (BD, Franklin Lakes, New Jersey, USA), ovarian hormone, thyroid hormone, and prolactin, folic acid and vitamin B12 (Roche Company, Basel, Switzerland), anti-phospholipid antibodies including anti-cardiolipin, anti-β2-glycoprotein, anti-phosphatidylserine/ ethanolamine (EUROIMMUN, Lubeck, German) and connective tissue antibodies including anti-dsDNA, Nucleosomes, Histones, SmD1, PCNA, Rib Po, SSA/Ro 60Kd, SSA/Ro 52Kd, SS-B/La, CENP-B, Scl 70, U1-snRNP, AMA M2, Jo-1, Pm-Sc1, Mi-2, Ku, and ANA (EUROIMMUN, Lubeck, German). All the patients were treated similarly according to their abnormal results except chromosomal problems. The treatment offered to pregnancy women with history of recurrent pregnancy loss (RPL): the RPL patients were offered progesterone or dydrogesterone, multivitamines or folic acids, vitamin E and low molecular heparin as preventive dose routinely. Levothyroxine was given according to patients’ thyroid stimulating hormone level. Low-dose aspirin, low molecule heparin and cyclosporin were added when the antiphospholipid antibodies were positive. Intravenous immunoglobulin and intralipid were administered to decrease NK cell activity. Granulocyte colony-stimulating factor (G-CSF) was applied when serum human chorionic gonadotropin rose slowly. The procedure of paternal lymphocyte immunization treatment (LIT): 20 ml peripheral blood was achieved from the husband and was diluted with equi-volume normal saline (NS), then mixed solution was added carefully to 15 ml lymphocyte separation liquids and centrifugated horizontally for 20 min at 400 g. The white cell layer was aspirated and washed with NS for two times. The cell precipitate was resuspended and injected into the subcutaneous tissue of wife’s cubitus. The LIT was applied three times before and two times after pregnancy every mouth for these primary RPL with negative blocking antibodies.

### Peripheral blood karyotype analysis

Chromosome analysis was performed on routinely cultured peripheral blood lymphocytes as described previously^12^. Briefly, the sections were treated with trypsin using standard techniques, the slides were Giemsa stained and then G-banding analysis was performed. Add colchicine 4 h before cytology preparation. For each sample, at least 20 cells from two independent cultures were used for microscopic observation and analysis in metaphase.

### Follow-up

All patients had been followed up for at least two years to get the subsequent first pregnancy outcomes. The details of each individual were entered into a computerized database with clinical features and miscarriage history recorded. Data of the present study was retrieved from medical records and telephone interviews.

### Statistical methods

t test was used for measurement data between the two groups, and chi-square test or Fisher’s exact test was used for count data. Binary logistic regression analysis was used to evaluate risk factors for pregnancy outcomes. *P* < 0.05 was considered statistically significant. The statistical software of SPSS 20.0 was applied in the study.

## Results

### The frequency and distribution of aberrant chromosomal RPL couples

**Figure 1:**
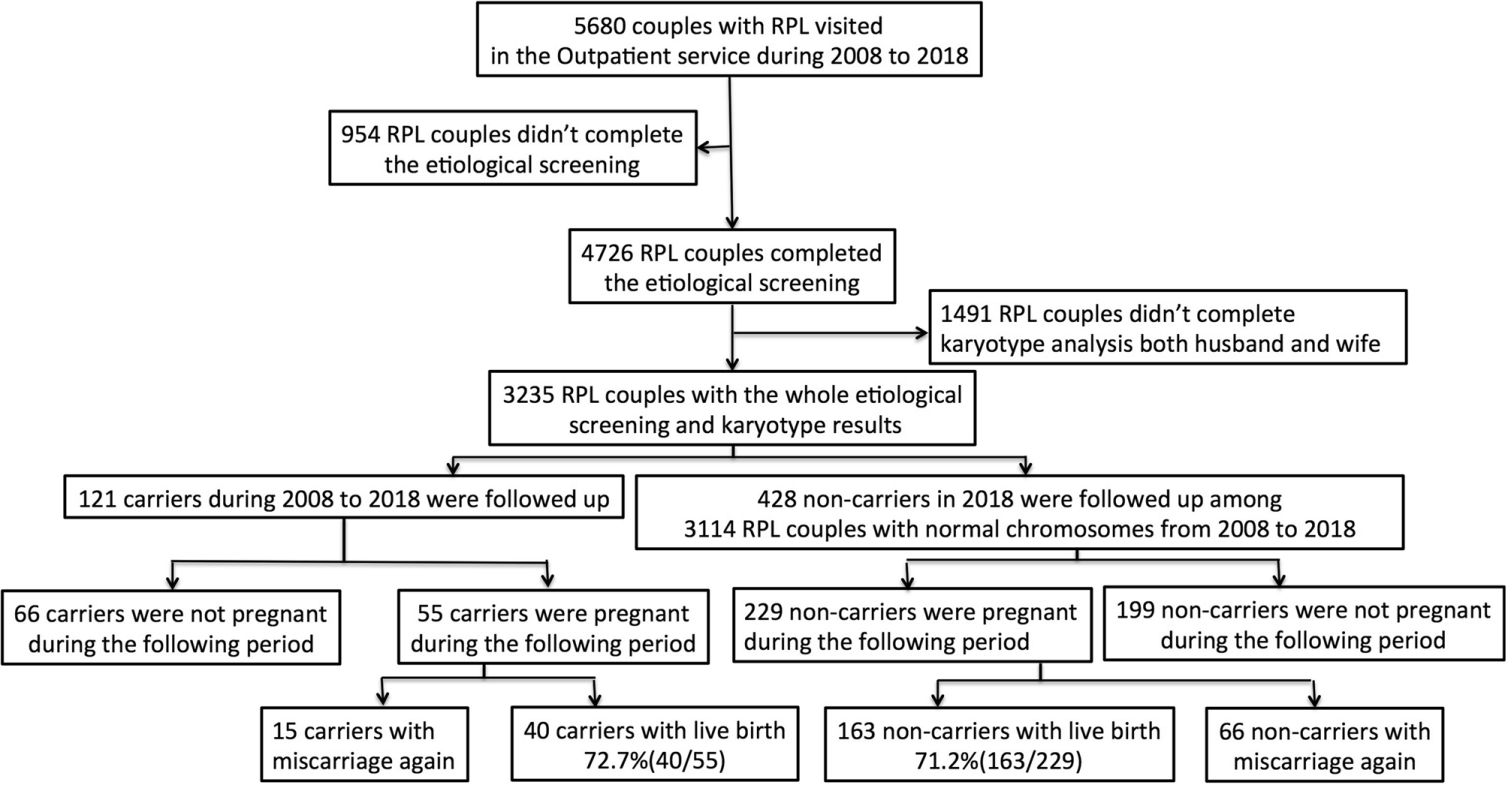
The flow chart presents the process of collecting abnormal chromosomal carriers from 5680 RPL couples in our Outpatient service.

The First Affiliated Hospital of Xi’an Jiaotong University is a tertiary referral teaching hospital. 5680 recurrent miscarriage couples came to the Reproductive Medicine Center from January 2008 to December 2018 as shown in Fig. 1. The flow chart shows that 954 couples had not completed the etiology screening evaluation and 1491 couples had not peripheral karyotype test of both female and male, so the remaining 3235 couples had complete karyotype analysis results. There are 121 couples of abnormal chromosomal karyotypes in 3235 couples with complete results (including abnormalities of either the female or the male and excluding chromosomal normal polymorphisms) with the abnormality rate of 3.74% (121/3235). Among 121 couples with abnormal chromosomal karyotypes, 101 cases were structural abnormalities (3.12%, 101/3235), and 20 cases were abnormal numbers (0.62%, 101/3235).

**Figure 2:**
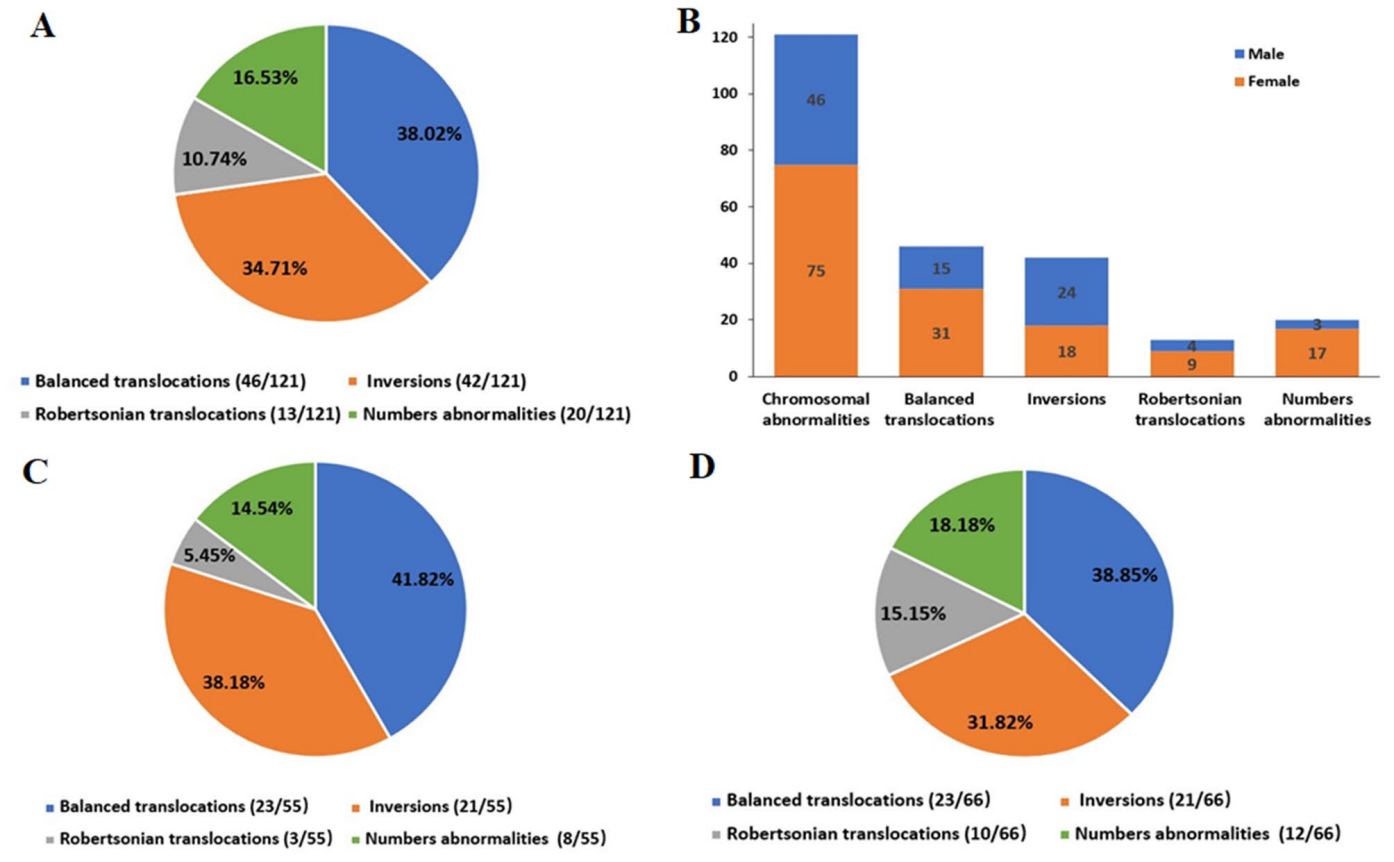
(**A**) The distribution of four aberrant types in the 121 RPL couples. (**B**) The respective numbers of female and male in the four aberrant type couples. (**C**) and (**D**). The distribution of four aberrant types in the 55 pregnancy and 66 non-pregnancy couples.

As shown in Fig. 2A, 101 structural abnormal cases included 46(38.02%) with balanced translocation, 42(34.71%) with inversion, 13(10.74%) with Robertsonian translocation, and 20(16.53%) cases had the numerical chromosome aberrations. In order to further illustrate whether the chromosomal abnormality comes from the female or the male, we noticed that 75 female and 46 male were with chromosomal abnormality among 121 RPL couples, and the distribution of abnormal chromosome types in female and male respectively can be shown in the Fig. 2B. During the following-up of 121 chromosomal abnormal couples with recurrent miscarriage, 55 couples were pregnant and 66 couples were not pregnant merely by medical expectant management through natural conception or intrauterine insemination without IVF/PGD as in the flowchart of Fig. 1. The proportions of the four types of chromosomal abnormalities among pregnant and non-pregnant carriers was shown in Fig. 2C and D. The two groups had no statistical difference in the four-type abnormal distribution by chi-square test (*P* = 0.31).

**Figure 3:**
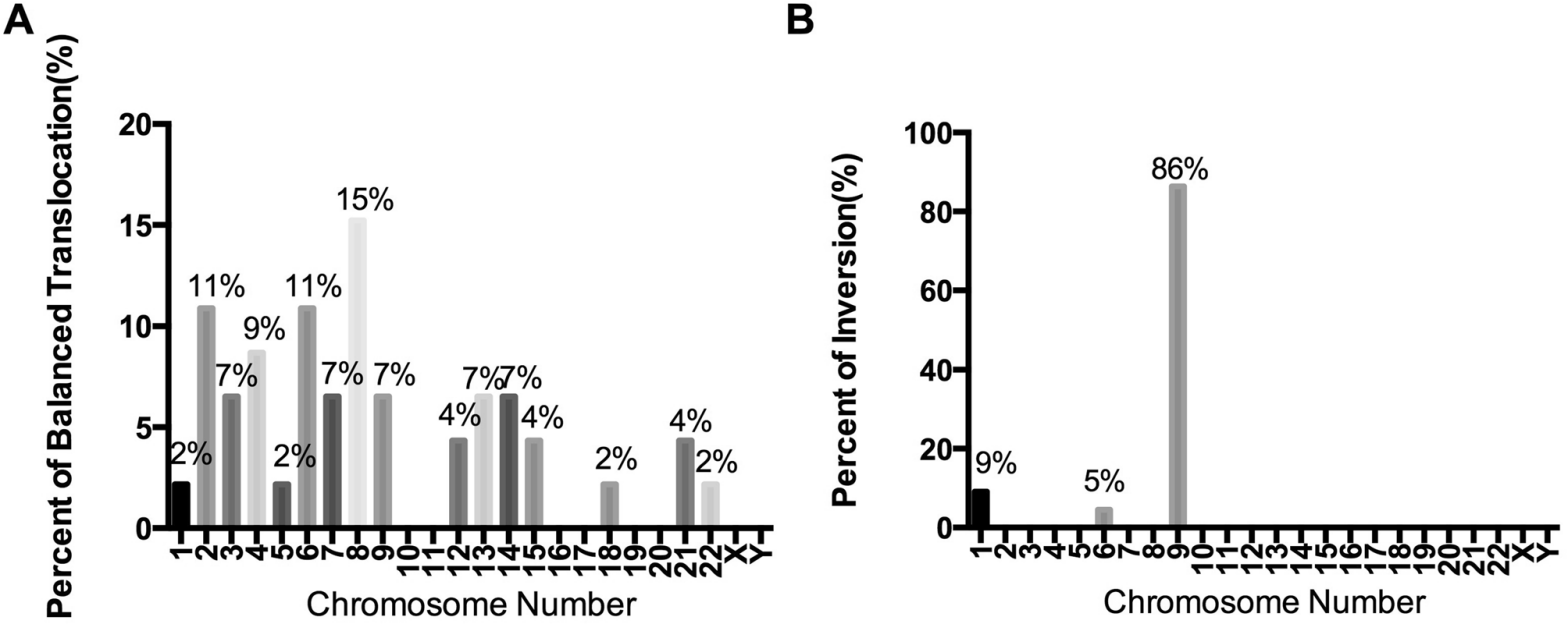
The percents of aberrant chromosome No. in the balanced translocation and inversion RPL patients. (**A**) for balanced translocation and (**B**) for inversion.

In the 55 carriers, the most common balanced translocation chromosome was No. 8 (15%) while the most rare types were No. 10, 11, 16, 17, 19, 20, X and Y (0%) shown in the Fig. 3A. The inversion of chromosome 9 accounted for 86%, the next was No.1 (9%) and No.6 (5%) as showed in the Fig. 3B.

### Comparison of the etiological results and live birth rates between 55 carriers and 229 non-carriers

Because it is difficult to achieve the complete the pregnancy results from thousands of patients from 2008 to 2018, 428 RPL couples with normal chromosomes who came to our outpatient department in the whole year of 2018 were selected and followed up for 2 years. They completed all etiological screenings and 229 of them were pregnant in the follow-up period as in the Fig. 1. Comparison of 55 carriers and 229 non-carriers showed that female carriers were younger at the time of consultation, while other clinical characteristics and combined pathological factors were not statistically different in Table 1. The outcome after the pregnancy, namely the live birth rate (LBR), was also not statistical different (*P* = 0.87). Among the 55 carriers, 51 carriers with primary recurrent miscarriage (no previous live birth history), and 4 were secondary recurrent miscarriages. 6 females were diagnosed with polycystic ovary syndrome (PCOS, according to the Rotterdam criteria^13^), and 3 females were diagnosed with decreased ovarian reserve (DOR, according to the hormonal markers and ultrasound parameters^14^). To further analyze of other etiological screening results in the 55 carriers, 8 cases (14.6%) were positive for infection factors (including male or female genital tract Mycoplasma and Chlamydia infection), 2 cases (3.64%) were with abnormal uterine anatomical structure, 14 cases (25.5%) were with imbalance of peripheral blood lymphocyte subsets, 9 cases (16.4%) were with endocrine disorders (including ovarian hormone abnormalities, thyroid hormone abnormalities and hyperprolactinemia), 7 cases (12.7%) were with nutritional element deficiency (including folic acid and vitamin B12). Among the combined autoimmune antibodies, 13 cases (23.64%) were positive for anti-phospholipid antibodies, and 9 cases (16.4%) were positive for connective tissue antibodies. During the follow-up period 40 in 55 pregnant RPL carriers gave birth to healthy babies in the way of natural conception or intrauterine insemination without IVF/PGD, the live birth rate (LBR) in the carriers (72.7%) was similar to that in the non-carriers (71.2%). It could be seen that, apart from age, the above-mentioned combined pathological factors and the final LBR was not statistically different between carriers and non-carriers in the Table 1. The results were still consistent after using binary logistic analysis to adjust the age factor.

**Table 1:** Analysis of combined non-genetic etiological factors and live birth rate of 229 RM non-carriers and 55 carriers.

	Non-carriers (n = 229)	Carriers (n = 55)	*P* value	Adjusted *P* value
Female age (years)	30.75 ± 3.89	29.13 ± 3.40	0.0049*	–
Number of previous abortions	2.42 ± 0.72	2.44 ± 0.63	0.865	0.912
Primary RM	84.3% (193/229)	90.9% (51/55)	0.18	0.807
PCOS	11.8% (27/229)	10.9% (6/55)	0.855	0.717
DOR	5.68% (13/229)	5.45% (3/55)	0.945	0.624
Infection	13.1% (30/229)	14.6% (8/55)	0.777	0.928
Anatomical uterine abnormalities	3.49% (8/229)	3.64% (2/55)	0.959	0.954
Lymphocyte subgroup abnormalities	24.5% (56/229)	25.5% (14/55)	0.877	0.553
Endocrine disorders	23.1% (53/229)	16.4% (9/55)	0.274	0.430
Nutrition abnormalities	14.8% (34/229)	12.7% (7/55)	0.688	0.490
APL Abs	34.9% (80/229)	23.6% (13/55)	0.109	0.086
Connective tissue Abs	10.9% (25/229)	16.4% (9/55)	0.264	0.324
Subsequent Live birth rate	71.2% (163/229)	72.7% (40/55)	0.819	0.968

**Figure 4:**
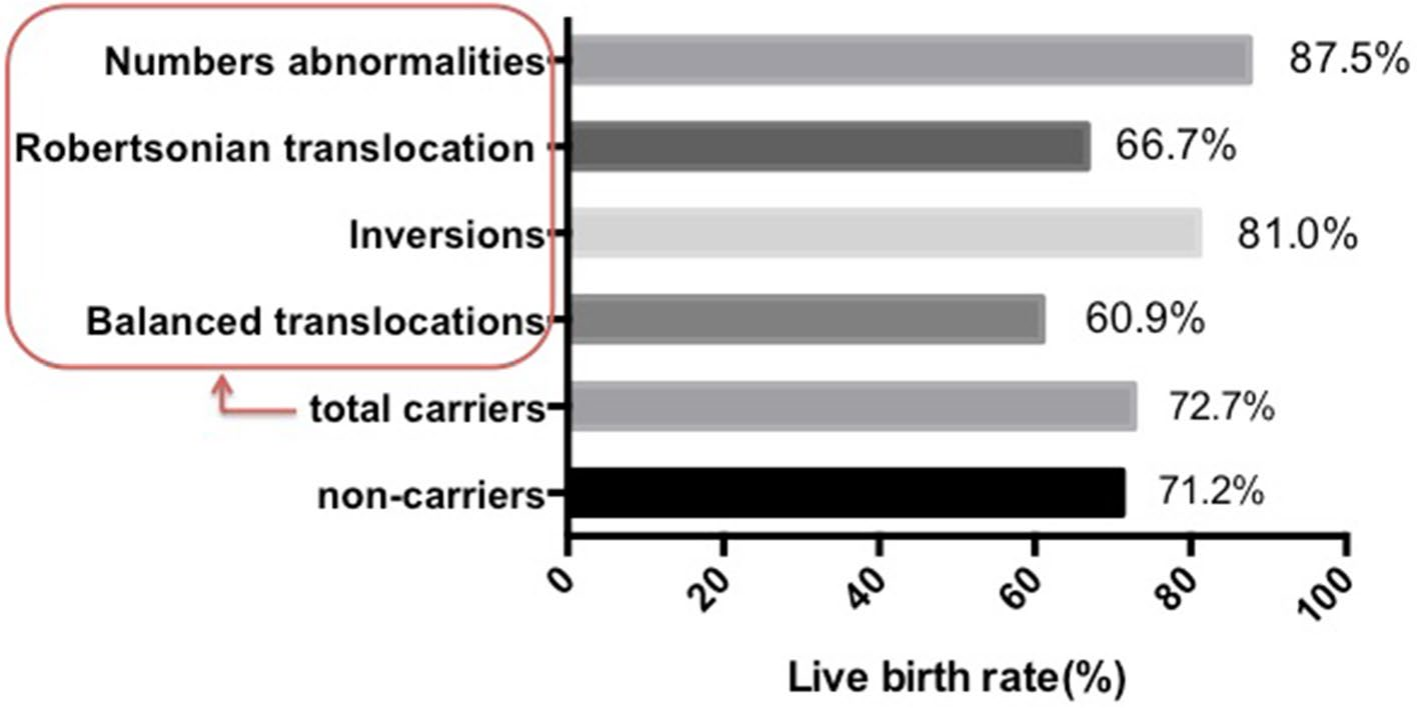
The live birth rates in the non-carriers and carriers of four aberrant types.

Table 2 showed the details of every patient number, the age of the female, the karyotypes of the female and the male, the number of miscarriages and the outcome of pregnancy of 55 carriers. 40 of 55 carriers gave birth to healthy newborns at the end with the LBR of 72.73%. Among the 40 cases, 7 cases were numerical abnormalities (LBR of 87.5%) and 33 cases were structural abnormalities (LBR of 70.21%). The structural abnormalities included 14 cases with balanced ectopic (LBR of 60.87%), 17 cases with inverted position (LBR of 80.95%), and 2 cases with Robertsonian translocations (LBR of 66.67%). As shown in Fig. 4, there was no statistical difference in the LBR in the four types of chromosomal abnormalities (*P* = 0.35).

**Table 2:** Detailed chromosome karyotype of 55 RM carriers and their pregnancy outcomes.

No	Age	Female chromosome	Male chromosome	No. of previous abortions	Pregnancy outcomes
1	25	46, XX, t (6; 8)	46, XY	2	Newborn health
2	25	46, XX, t (6; 7)	46, XY	2	Newborn health
3	25	46, XX, t (2; 6)	46, XY	4	Newborn health
4	26	46, XX, t (8; 12)	46, XY	2	Newborn health
5	28	46, XX, t (4; 13)	46, XY	2	Newborn health
6	28	46, XX	46, XY, t (5; 7)	2	Newborn health
7	31	46, XX, t (6;18)	46, XY	2	Newborn health
8	27	46, XX, t (4; 14)	46, XY	2	Newborn health
9	24	46 XX, t (2; 3)	46, XY	4	Newborn health
10	32	46, XX, t (8; 9)	46, XY	2	Newborn health
11	28	46, XX, t (6; 9)	46, XY	2	Newborn health
12	30	46, XX, t (14; 21)	46, XY	3	Newborn health
13	27	46, XX, t (2; 4)	46, XY	4	Newborn health
14	30	46, XX	46, XY, t (4; 21)	2	Newborn health
15	26	46, XX	46, XY, t (14; 22)	2	7 W, miscarriage
16	29	46, XX, t (3; 13)	46, XY	2	13-trisomy, odinopoeia
17	28	46, XX, t (8; 15)	46, XY	3	7 W, miscarriage
18	29	46, XX, t (8; 9)	46, XY	3	8 W, miscarriage
19	24	46, XX	46, XY, t (1; 8)	2	6 W, miscarriage
20	30	46, XX, t (8; 15)	46, XY	2	8 W, miscarriage
21	23	46, XX, t (3;13)	46, XY	3	13- trisomy, odinopoeia
22	27	46, XX	46, XY, t (2, 12)	2	20 W, miscarriage
23	31	46, XX, t (2; 7) inv (9)	46, XY	4	7 W, miscarriage
24	30	46, XX, inv (9)	46, XY	3	Newborn health
25	28	46, XX	46, XY, inv (9)	2	Newborn health
26	28	46, XX, inv (9)	46, XY	2	Newborn health
27	35	46, XX	46, XY, inv (9)	3	Newborn health
28	31	46, XX, inv (9)	46, XY	3	Newborn health
29	29	46, XX	46, XY, inv(1)	2	Newborn health
30	26	46, XX, inv (9)	46, XY	2	Newborn health
31	30	46, XX	46, XY, inv (9)	2	Newborn health
32	27	46, XX	46, XY, inv (9)	2	Newborn health
33	31	46, XX, inv (6)	46, XY	3	Newborn health
34	30	46, XX	46, XY, inv (9)	2	Newborn health
35	28	46, XX, inv (9)	46, XY	2	Newborn health
36	35	46, XX, inv (1)	46, XY	2	Newborn health
37	37	46, XX, inv (9)	46, XY	2	Newborn health
38	28	46, XX	46, XY, inv (9)	2	Newborn health
39	32	46, XX	46, XY, inv (9)	3	Newborn health
40	28	46, XX	46, XY, inv (9)	3	Newborn health
41	31	46, XX	46, XY, inv (9)	2	6 W, miscarriage
42	29	46, XX	46, XY, inv (9)	2	14 W, miscarriage
43	33	46, XX, inv (9)	46, XY	2	7 W, miscarriage
44	31	46, XX	46, XY, inv (9)	2	21 W, miscarriage
45	27	45, XO	46, XY	2	Newborn health
46	28	47, XX, +mar	46, XY	3	Newborn health
47	38	46, XX	46, XY/46, XX(5%)	3	Newborn health
48	35	46, XX [47]/45X, [3]	46, XY	3	Newborn health
49	24	46, XX	47, XY, +mar	3	Newborn health
50	38	45, X[3]/46, XX[57]	46, XY	3	Newborn health
51	30	45, X[3]/46, XX[97]	46, XY	2	Newborn health
52	30	46, XX	47, XY, +mar	2	8 W, miscarriage
53	28	46, XX	45, XY, rob (13; 14)	2	Newborn health
54	27	45, XX, rob (13; 14)	46, XY	2	Newborn health
55	27	45, XX, rob (13; 14)	46, XY	3	7 W, miscarriage

Among the 55 pregnant couples, 34 were female and 21 were male carriers. In the Table 3 we analyzed the women’s age, number of miscarriages, distribution of karyotype abnormalities and the total LBR in female and male carriers respectively. There was no statistical difference in all items and showed gender of carrier had no effect on the pregnancy outcome (*P* = 0.428).

**Table 3:** Pregnancy outcomes of 34 RM couples with female carriers and 21 RM couples with male carriers.

	Female carriers (n = 34)	Male carriers (n = 21)	*P* value
Female age (years)	28.97 ± 3.01	29.09 + 2.72	0.879
No. of previous abortions	2.54 ± 0.71	2.24 ± 0.44	0.096
Numerical abnormalities	100.00% (5/5)	66.67% (2/3)	0.167
Abnormal chromosome structures	72.41% (21/29)	66.67% (12/18)	0.675
Balanced translocations	66.66% (12/18)	40.00% (2/5)	0.279
Inversions	88.89% (8/9)	75.00% (9/12)	0.423
Robertsonian translocations	50% (1/2)	100% (1/1)	0.386
Total success rates	76.47% (26/34)	66.67% (14/21)	0.428

In order to rule out the influence of other etiological factors on the pregnancy outcomes, we analyzed the female age, the number of abortions, infection factors, anatomical uterine abnormalities, autoimmune antibodies positive rate, blocking antibody deficiency, peripheral blood lymphocyte subset disorders, endocrine disorders and nutritional elements deficiency between 40 carriers with live birth and 15 carriers with miscarriage again in the Table 4. All the differences in above items between the two groups were not statistically significant and no one showed huge influence to alter pregnant outcomes.

**Table 4:** Other relevant causes of 40 RM couples with live birth and 15 RM couples with miscarriage again.

	Carriers with live birth (n = 40)	Carriers with miscarriage again (n = 15)	*P* value
Female age (years)	29.35 ± 3.62	28.40 + 2.67	0.359
No. of previous abortions	2.43 ± 0.64	2.40 ± 0.63	0.897
Infection	15.00% (6/40)	6.67% (1/15)	0.658
Anatomical uterine abnormalities	2.50% (1/40)	6.67% (1/15)	0.475
Autoimmune antibodies	52.50% (21/40)	60.00% (9/15)	0.764
Blocking antibody deficiency	75.00% (30/40)	53.33% (8/15)	0.122
Lymphocyte subgroup abnormalities	27.50% (11/40)	33.33% (5/15)	0.671
Endocrine disorders	12.50% (5/40)	13.33% (2/15)	0.428
Nutrition abnormalities	22.50% (9/40)	26.67% (4/15)	0.746

## Discussion

The cause of spontaneous abortion is generally attributed to two sources, namely seed or gamete problems and environmental problems. Gamete problems are often considered to be abnormal parental chromosomes or abnormal fetal chromosomes. The results of this study showed that the incidence of chromosomal abnormalities in couples with recurrent miscarriage was 3.74% (Fig. 1). The present results consistent with previous studies have shown that the incidence of chromosomal abnormalities in the general population is less than 1%^15,16^ and RPL population is 2–5%^17,18^, indicating that parental chromosomal abnormalities rate increased assuredly in the miscarriage couples.

Balanced translocation was the most common type, accounting for 38.02% and consistent with other findings^11^. A meta-analysis from Zouhair reported that frequency of chromosomal abnormalities in couples with RPL was 5.16% and the most common reciprocal translocation accounts 48.4% in the worldwide literature review^12^. The balanced translocations and inversions will not affect the parents themselves in phenotype, but their unbalanced gametes during meiosis may indeed be part of the cause of miscarriage. Similarly, Robertsonian translocation of parental chromosomes can also cause miscarriage, birth defects or mental retardation of offspring^19^. However, all these studies could not demonstrate the explicit causality between aberrant chromosome and abortions.

Additionally, the LBR of RPL carriers in our reproductive center have reached more than 70%, indicating that the proportion of miscarriage caused by chromosomal abnormalities in RPL couples was very slight. A retrospective study from Howard et al. concluded that no statistically significant was found in the LBR between RPL couples with chromosomal abnormality (45.2%, 33/73) and the normal couples was (55.3%, 325/588), regardless of number of miscarriages and rearrangement types of chromosomal abnormalities^20^. In Goddijn's study the screening results of 1324 RPL couples showed that all the 41 couples with abnormal structure chromosomes did not yield an unbalanced fetal chromosome pattern^21^. It is also consistent with Franssen’s study, the LBR of RPL carriers was equivalent to the normal couples after six accumulated gestations, and had no relevance with the type of abnormal chromosome (83% vs. 84%)^22^. However, Sugiura’s study showed pregnancy prognosis was worsened with either maternal or paternal reciprocal translocations than normal couples (63% vs. 78.7% of LBR)^23^. Pregnancy outcomes for RPL couples with chromosomal abnormalities were still very satisfactory generally, although the decrease in the live birth rate may not have been detected due to insufficient sample size in our study. In addition, inversion of chromosome 9(inv(9)) is also considered as normal polymorphism in other reports^24,25^. The live birth rate of inv(9) in our study is 77.8%(14/18) which is not significantly different with the LBR in the other groups.

Preimplantation genetic testing (PGD) has been proposed as a controversial method in the worldwide for selecting normal chromosome embryos in the IVF to lower risk of miscarriage for patients with unexplained RPL and balanced translocations carriers. However, well-designed trials comparing EM (expectant management) to PGD have not been performed. Several previous cases indicated benefits of PGD including fewer miscarriages and shorter time to successful pregnancy without taking into account the emotional and financial cost of a failed or canceled cycle. More recent reports suggested clinical outcomes including pregnancy rate, live birth rate (53% vs. 67%) and clinical miscarriage rate were similar between PGD and EM among recurrent miscarriage patients^26^. Even in the parental carriers of structural chromosomal rearrangement and history of RPL, no significant difference with regards to reproductive outcomes such as miscarriage rate, time to live birth, or live birth rate was observed between couples who pursued PGD compared with EM^27,28^. These data combined with our results allow us to reflect on the actual benefits of PGD to these patients, so clinicians can be more cautious when making an alternative of PGD in clinical work. Natural conception is also recommended as a good alternative for these aberrant chromosomal carriers.

50–60% of spontaneously aborted product of conception have been detected with chromosomal abnormality^29^. The abnormal chromosomes of the fetus are derived from the parental abnormal chromosomes or produced in the process of gamete meiosis and mitosis of the fertilized egg by mistake randomly. The types of fetus abnormality were often mainly manifested as trisomies of chromosome 13, 18, 21 and X monosomy (45, X)^30^, but not consisted with the translocation chromosomes of the parents showed as in the Fig. 3. According to Howard Carp’s study, parental karyotyping was not particularly predictive of a subsequent miscarriage, 43.5% of abortus from parental carriers were euploidic and the parental aberration was passed on to the abortus in only 10% of cases^31^. The phenotypes are inconsistent that parental karyotyping prefers balanced translocations (No. 8, 2, 6) and inversions (No. 9, 1, 6) rather than the more common numerical aberrations such as trisomies (No. 13, 18, 21) and polyploidy in fetus. Most aberrant chromosomes in the fetus are generated randomly and only a small percent derives from their parents.

One of the most important results in our data is the influence of parental chromosomes on live birth rate (LBR). In our study, the LBR of both carriers and non-carriers can reach about 70% without relationship of gender, female age, chromosome abnormal type, number of previous abortions and other pathological factors. Amounts of non-genetic pathological causes related to endocrine, infection, immune and nutrition were detected not only in aberrant chromosomal carriers but also in non-carriers, while these factors have a strong impact on the pregnancy outcomes. After effective treatments such as anticoagulation and immunotherapy, the LBR of re-pregnancy after two recurrent miscarriages has reached more than 70% internationally. A prospective study showed that closely following management and treatment of other high-risk factors can increase the LBR of RPL couples with chromosomal abnormalities from 25 to 70%^32^ or from 20 to 71% without the addition of assisted reproductive technology^11^. The differences of LBR in RPL carriers in previous reports may due to the different management of non-genetic pathological factors that are usually more important in fetal survival.

The formation frequency of abnormal gametes theoretically is not equal to the birth rate of abnormal babies in practice. We still recommend that the chromosome test or next-generation sequencing analysis of the amniotic fluid through puncture should be performed around 18 weeks of gestation in the natural pregnancy of RPL patients with chromosomal abnormalities, even so the deletion or duplication of smaller fragments still cannot be detected.

We strongly recommend that RPL carriers should still undergo comprehensive and systemic etiological screening. It is necessary to actively deal with other causes of miscarriage in order to improve the chances of successful pregnancy for RPL patients with chromosomal abnormalities. In order to improve the live birth rate, our treatment included surgical correction of the anatomically abnormal uterus, paternal lymphocyte treatment, anticoagulation aimed at anti-phospholipid antibody and immunosuppressive therapy were strongly recommended besides chromosome abnormality in our opinions. Paternal lymphocyte treatment and immunosuppressive therapy were done according to our experience and suggestions from some published reports^33–35^. However, many of the treatments offered to patients with recurrent pregnancy loss especially unexplained cause are not based on good evidence. A comprehensive reviews showed there was no role for immunotherapy in improving the LBR in women in the prevention of idiopathic RPL^36^.

The present study did not detect the karyotype of aborted fetuses and not achieve complete amniocentesis results from the pregnancy carriers. So we could not assess the impact of fetal chromosomal problems came from parents. In the study of embryo chromosome analysis of abortion tissue, trisomy and polyploidy are the majority which account for 65% and 17% respectively, a considerable proportion of fetus with aberrations include trisomy, structural abnormality and low-frequency mosaic could survive after birth^30^. The phenotypes are inconsistent that parental karyotyping prefers balanced translocations and inversions rather than the more common numerical aberrations such as trisomy and polyploidy in fetus. In addition, G-banding karyotype analysis used in this study can only detect a part of patients with abnormal numerical and structural chromosomes. Conventional karyotype analysis identifies balanced and unbalanced chromosomal rearrangements and copy number variants (CNVs) to a ∼ 5 Mb resolution. Due to the limitations of the detection method itself, it could not exclude some other types of genes or chromosome abnormalities related to miscarriage problems, such as deletion, insertion, duplication and point mutation of some gene fragments. In 2019, Chen et al. used low pass genome sequencing (GS) to detect the chromosomes of RPL couples with abnormalities rate increased to 11.7% compared to traditional karyotyping with 5.7%. However, inversions and copy-number variants detected by GS additionally had not been confirmed to directly related with miscarriage. 10 carriers observed in follow-up observations and five of them miscarried again (miscarriage rate of 50%). The small sample size did not indicate that the risk of miscarriage of abnormal chromosome couples was higher than that of couples with normal chromosomes^37^.

Finally, the lack of samples even in this 11-year study and other combined known and unknown non-genetic factors are shortcomings in the present data. The etiology of recurrent miscarriage is complicated and there are many controversies in the treatment. The coexistence of these other pathological factors and chromosomal abnormalities makes the results confused and controversial.

## Conclusions

In conclusion, balanced translocation is the most common phenotype in RPL carriers, and LBR of subsequent first pregnancy is similar to the non-carriers. The present studies can help to provide more scientific clinical consultation, such as more accurate diagnosis and the prognostic outcome of subsequent pregnancy, and help doctors to raise awareness of miscarriage-related chromosome problems and foster a theoretical basis for reasonable treatment.
